# Supplementary material for: Impact of YAQ001 on the Gut Microbiome, Permeability and Markers of Systemic Inflammation in Patients With Cirrhosis
Source: Liver Int. 2026 Oct 21;46(11):e70933. doi: 10.1111/liv.70933 (PMC13686283; doi:10.1111/liv.70933)
Supplement: Supplementary file 1 [file LIV-46-0-s001.zip › YAQ001 Supplementary Data Final.docx]

**Impact of YAQ001 on the gut microbiome, permeability and markers of systemic inflammation in patients with cirrhosis**

Jinxia Liu, Fernando Garcia-Guevara……Rajiv Jalan

**Table of contents**

Supplementary Methods--------------------------------------------------------------------------2

Fig.S1-------------------------------------------------------------------------------------------------10

Fig.S2-------------------------------------------------------------------------------------------------11

Fig.S3-------------------------------------------------------------------------------------------------12

Fig.S4-------------------------------------------------------------------------------------------------13

Fig.S5-------------------------------------------------------------------------------------------------15

Fig.S6-------------------------------------------------------------------------------------------------16

Fig.S7--------------------------------------------------------------------------------------------------17

Fig.S8--------------------------------------------------------------------------------------------------17

Fig.S9--------------------------------------------------------------------------------------------------17

Supplementary Tables and legends-----------------------------------------------------------18

Supplementary references-----------------------------------------------------------------------22

**Supplementary Methods**

**Core laboratory analyses**

***Luminex Assay:*** The Luminex Assay Protocol was performed for measurement of systemic cytokines in accordance with the manufacturer’s instructions. In brief, 50 mcl of sample/standard and 50 mcl of Microparticle Cocktail are added to a 96 well plate, sealed and incubated for 2 hours at room temperature on a horizontal orbital microplate shaker. The plate was then washed three times with wash buffer and 50 μL of diluted Biotin Antibody Cocktail added to all wells. The plate was sealed and incubated for 1 hour at room temperature. 50 μL of Streptavidin- Phycoerythrin (PE) was then added to all wells and incubated for 30 minutes at room temperature. Following washing with buffer, the microparticles were then resuspended in Wash Buffer and incubated for 2 minutes at room temperature. The plate was then read within 90 minutes using Bio-Plex® MAGPIX™ Multiplex Reader and analysed on xPONENT software.

Faecal cytokine analysis and D-lactate assays were performed as previously described.^1^

***Bile Acids (BAs) Analysis:*** Cholic acid (CA), chenodeoxycholic acid (CDCA), deoxycholic acid (DCA), lithocholic acid (LCA), ursodeoxycholic acid (UDCA) and their glycine and taurine conjugated species were identified and quantified by ultra-performance liquid chromatography - quadrupole time-of-flight mass spectrometry (UPLC/Q-TOF-MS). Mass spectrometry (MS) analysis was performed using Xevo G2-XS Qtof system from Waters using an electrospray interface. BAs were separated by elution gradient mode with a mobile phase composed of a mixture of ammonium acetate buffer 15 mM, pH 8.0 (Solvent A) and acetonitrile: methanol = 75:25 v/v (Solvent B). The separation was obtained with a total run time of 6 minutes. All the chromatograms were acquired in electrospray negative ionization with the mass spectrometer operating in multiple reactions monitoring mode.

***Analysis of Short-chain fatty acids (SCFAs) in stool:*** Prior to analysis, Eppendorf tubes were weighed and approximately 100 mg of stool was added to individual tubes. These stool samples were then homogenized in 1mL of buffer (0.1 M Tris, 0.15 M NaCl, 1 M urea, 10 mM CaCl2, 0.1 M citric acid monohydrate, 5 g/L bovine serum albumin (BSA) and 0.25 mM thimerosal, pH 8.0) using a plastic homogenizer.

***Sample preparation and derivatization:*** 20 mL of stool sample and 15 mL of Internal standard mix was added to a fresh glass vial. The volume in the vial was adjusted to 100 mL with d.H2O. 500 mL of 0.1M tetrabutylammonium (TBA) bisulfate in 0.1 M KH2PO4 (pH 7.4) was added to each vial followed by 500 mL of 2% Pentafluorobenzyl bromide (PFBBR) in dichloromethane. Samples were then sonicated for 60 minutes. Samples were extracted into 2 mL of hexane and centrifuged at 1500 rpm for 5 minutes at 4°C. The organic phase was moved to a fresh glass vial and evaporated under N2 gas to approximately 100 mL. 100 mL of sample was then transferred to a glass autosampler vial and run via Gas Chromatography-Mass Spectrometry (GC-MS). Samples were analysed on Rxi®-5Sil MS fused silica, 30 m x 0.25 mm I.D, 0.25 µm film thickness), Inlet Temperature: 280°C, MS Transfer line: 250°C, Split ratio: 1: 10, carrier gas (Helium) flow rate: 0.9 mL/min, ion source: 225°C, chemical ionization (Methane 2 mL/min). Oven temperature was programmed from 45°C to 200°C at 10°C/min, then to 300°C at 30°C/min. Negative ions were detected in selected ion monitoring: The remaining stool sample was dried overnight using a vacuum centrifuge and dry weight was calculated.

**Intestinal sugar permeability tests**

Urine samples (300ml plus 100ml internal standard mix ([^13^C_6_-glucose [Sigma Aldrich]+ ^13^C_12_-sucrose [Carbosynth]) added to 50mg washed and dried mixed bed resin (Amberlite MB 150 Mixed Bed Exchanger, Sigma), which was then vortexed and centrifuged (13,000 rpm x 5min). 200ml supernatant was dried under N_2_, derivatized with 10ml methoxyamine (Alfa-Aesar) in dry pyridine (Sigma Aldrich) (50mg/ml) and incubated for 90 minutes at 30^o^C. 90 ml *N*-methyl-*N*-trimethylsilyl)trifluoro-acetamide with 1% trimethylchlorosilane were added and incubated for 30 minutes at 37^o^C.

Samples were analysed by GC/MS (Agilent 5973MSD with 5973 GC), using a Rxi®-5Sil MS fused silica (30m x 0.25mm I.D, 0.25µm film thickness, with 10m guard column; Thames Restek) with a helium flow of 1.3ml/min. 2µl were injected at a 25:1 split ratio (inlet temperature 250 ^o^C) and oven temperature starting at 150 ^o^C, held for 1 minute, and then ramped to 325 ^o^C at 5 ^o^C/min. Transfer line was held at 300 ^o^C, and sugar-trimethylsilyl oximes were detected in scan mode. Retention times and quantification ions were as follows: xylose 7.2-7.5 min m/z 307.2, rhamnose 8.4-8.5 min m/z 277.2, [U-^13^C_6_]glucose 11.5min, m/z 323.2, [U-^13^C_12_] sucrose 23.8min m/z 367.2, lactulose 24.5-24.7 m/z 361.2. Xylose and rhamnose were quantified as peak area ratios relative to the peak area of [U-^13^C_6_] glucose, and lactulose as peak area ratio relative to the peak area of [U-^13^C_12_] sucrose.

**Creatinine**

Urine samples were corrected to creatinine to account for urine output. Urine creatinine was measured using a quantitative enzymatic colorimetric assay kit (Sentinel Diagnostics) according to the manufacturer’s instructions, on a Tecan Infinite F-200 plate reader at 555nm.

**Nuclear Magnetic Resonance (NMR) analysis**

Aliquots of plasma (from blood collected into lithium heparin tubes) and urine were stored frozen at -80^o^C until NMR analysis. All samples were gently thawed in preparation for NMR analysis at the Biomolecular NMR Centre, King’s College London. Urine and serum samples were prepared for NMR analyses using standardised protocols. NMR data were acquired at the Centre for Biomolecular Spectroscopy, King’s College London using a Bruker 600 MHz (AVANCE NEO) NMR spectrometer and a 1H/13C/15N TCI Prodigy (nitrogen-cooled) probe. Pulse-collect (urine, serum) and spin-echo (serum) 1D NMR data sets were acquired using Presaturation Utilizing Relaxation Gradients and Echoes (PURGE) water suppression and Periodic Refocusing of J Evolution by Coherence Transfer (PROJECT) spin-echo sequences.

**Analysis of alterations on the microbiome**

*DNA extraction*

For fecal samples, 200 mg of pre-homogenized material was processed using a modified International Human Microbiome Standards (IHMS) protocol.^2^ Samples underwent enzymatic lysis (lysozyme, 37°C, 30 min), mechanical disruption (bead-beating, 0.1 mm glass beads, 5 min), and chemical digestion (Proteinase K, 65°C, 1 h). DNA was purified via silica-column binding, washed, and eluted in 100 μL nuclease-free water. Concentration was measured by Qubit, and quality was verified by 16S ribosomal RNA (rRNA) polymerase chain reaction (PCR) and electrophoresis. Extraction blanks and positive controls were included for quality assurance.

*Metagenomic sequencing*

Stool samples were subjected to metagenomic sequencing using the Illumina HiSeq 2000 platform (Illumina, San Diego, CA), generating paired-end reads (2×100 bp). After quality filtering, each sample yielded a minimum of 10 million reads, with an average of 12.5 ± 2.1 million reads (mean ± SD). Raw sequencing data were assessed using FastQC v0.11.3,^3^ with all metagenomes passing >50% of the predefined quality metrics. Quality reports were aggregated and visualized using MultiQC,^4^ confirming that all samples met the following stringent criteria: base quality scores (Q30 > 85% of bases); uniform read length distribution (100 ± 2 bp); stable GC content (45 ± 5%); minimal adapter contamination (< 0.1%); low duplicate rate (< 5% of reads). Only high-quality reads fulfilling these standards were retained for downstream bioinformatic analyses.

*Gene and Species abundance profiling.*

The raw reads for all samples were trimmed using Alien Trimmer 0.4.0 with parameters -k 10 -l 45 -m 5 -p 40 -q 20 and Illumina contaminant oligonucleotides.^5^ Trimmed samples were mapped against the Integrated Gut Catalogue 2 (IGC2)^6^ using Bowtie integrated into the Metric for Evaluation of Translation with Explicit ORdering (METEOR) pipeline (https://forgemia.inra.fr/metagenopolis/meteor) against a catalogue that contains Metagenomic Species Pan-genomes (MSPs) of the human gastrointestinal microbiota built by binning co-abundant genes of the IGC2^7,8^ catalogue with MSP miner.^9,10^ Human contaminant sequences were removed from all samples by discarding reads that mapped against a human reference genome. Downsizing of the gene count tables was done using the MetaOMineR (MOMR) downsize and a cutoff of 10 M reads. Matrix function was used to eliminate the sequencing depth fluctuations. Normalization of the downsized gene count tables was performed using the MOMR norm frequency Reads Per Kilobase of exon model per Million mapped reads (FreqRPKM) function and according to the fragments per kilobase of transcript per million fragments mapped (FPKM) strategy. MSP abundances were estimated using mean abundance of 50 ‘marker’ genes (highly correlated within the MSP). Less than 10% of ‘marker’ genes were assumed to have species abundance of 0.

*Functional annotation of Genes in IGC2 catalogue.*

Protein sequences of the IGC2 catalogue were annotated for Antibiotic Resistance Genes using the DeepARG software,^11^ using a minimum probability cutoff of 0.9. Proteins of IGC2 catalogue were annotated to Kyoto Encyclopedia of Genes and Genomes (KEGG) orthologs using Diamond against KEGG database.^12^ Best-hit alignments with e-value < 10e-5. Annotation of VFs was performed using blastp Best-hit alignments with an e-value cutoff of 1e10-10 and a minimum percentage identity of 65% against the set of sequences provided in the virulence factor database (VFDB).^13^ Annotation of Secondary BA genes was performed using best-hit alignments of blastp running against sequences of genes and suggested cutoffs as previously reported (BaiA, HDHA, BaiL, BaiB, BaiCD, BaiH, BaiF, BaiK, BaiG, e-value 1e-10; BaiJ, e-value 1e-5).^14^ To construct the KO, VF, and ARG abundance tables, we started from the gene count table, grouped genes sharing the same annotation (KO, VFID, or ARG), and summed their abundances.

**Biofilm prevention and antibiofilm activity of YAQ001 against K. pneumonia TOP52**

*K. pneumonia* TOP52 was pre-cultured statically in TSB broth at 37 °C for 48 h, diluted to OD_600_ of 0.1 in fresh medium, and inoculated into 24-well polystyrene plates (1 mL/well). Plates were incubated statically at 37 °C. Biofilm biomass was quantified at 24, 48, and 72 h by crystal violet staining (Sigma-Aldrich). Wells were gently washed with PBS, stained with 0.1% CV for 20 min at room temperature, washed three times with PBS, air-dried, and solubilized in 33% acetic acid (Fisher); absorbance was measured at 595 nm using a Tecan Spark microplate reader. For inhibition assays, YAQ001 particles (0.625–10 mg/mL in TSB) were added at the time of inoculation in a total volume of 1 ml, and biofilms were incubated for 72 h. For antibiofilm assays, mature biofilms were pre-formed for 48 h before adding YAQ001 as above, followed by an additional 14 h treatment. Controls included untreated Yaq-001 (PBS only), antibiotic controls meropenem (ApexBio) and ciprofloxacin (ThermoFisher) in PBS alone and in combination with 10 mM EDTA. Biofilms were treated with antibiotics from 1× to 10000× MIC in both biofilm prevention assay and antibiofilm assays. 3 independent experiments were conducted.

*Effect of YAQ001 on K. pneumonia TOP52 infection in a polarized human intestinal model.*

A polarized human intestinal epithelial layer was established by co-culturing Caco2 colorectal epithelial cells and HT29-MTX-E12 mucous-producing, goblet-like colorectal cells at a 3:1 ratio on Snapwell inserts (12 mm diameter, 0.4 µm pore size; Corning) for 14 days in DMEM supplemented with 10% FBS, 1% non-essential amino acids, and 1% penicillin–streptomycin. Medium was refreshed every 2–3 days until maturation. For infection, K. pneumoniae TOP52 (GFP-tagged) was grown statically in TSB supplemented with spectinomycin (200 µg/ml) at 37 °C for 48 h. Next, the culture was adjusted to an OD_600_ of 0.001 or 0.005 corresponding to approximately 1×10^6^ CFU/ml and 2 ×10^6^ CFU/ml in DMEM antibiotic-free medium for the infection. Next, the apical chamber was infected using DMEM antibiotic-free medium for 14 h at 37 °C. For treatment, YAQ001 particles (10 mg/mL in PBS) were applied simultaneously with bacteria to a total volume of 500µl. Untreated control (PBS) was included for comparison. After 14h, the apical side (containing planktonic bacteria) was carefully collected, ensuring minimal disruption to the mucus layer. To ensure full recovery of remaining bacteria, the apical compartment was washed with an equal volume of sterile PBS, which was pooled with the original sample. Planktonic fractions were serially diluted (log₁₀) and plated on LB agar plates (Sigma-Aldrich) in 10 µL spots for CFU enumeration the next day. 3 independent experiments were conducted in this study.

*Confocal Microscopy and immunofluorescence staining*

For fixation and imaging, co-cultures were fixed with 4% paraformaldehyde (PFA; Invitrogen) for 15 minutes at room temperature. Following fixation, membranes were cut from snapwells (corning) using a scalpel and blocked with 3% BSA in PBS (Merck) for 30 minutes. For permeabilisation, the membrane was submerged in 0.2% Triton X-100 (Sigma-Aldrich) for 20 minutes. Detection of actin and nuclei was stained with Phalloidin (Invitrogen) conjugated to Alexa Fluor 647 (1:200 dilution in PBS) and DAPI (Invitrogen), respectively (1:1000 dilution in PBS) for 30 minutes. Membranes were mounted with ProLong Glass Antifade mountant (Invitrogen) onto glass slides, and visualization was performed with a Leica SP8 confocal laser scanning microscope. Images were processed using the Leica Application Suite (LASX).

*Minimum inhibitory concentration assay*

Minimum inhibitory concentrations (MIC) assays were determined according to the standard Clinical Laboratory Standards Institute (CLSI) broth microdilution method (M07-A9-2012) using cation-adjusted Müller-Hinton broth (caMHB). Overnight cultures of bacteria *K. peumoniae* TOP52 were performed in caMHB in shaking conditions at 37 °C. The concentration of bacterial cells was adjusted by measuring the optical density at 600 nm (OD_600_) to obtain an equivalent to a McFarland standard of 0.5 (OD_600_ ∼ 0.08−0.1), corresponding to a bacterial concentration of ∼10^8^ colony-forming units per mL (CFU/ml). The solution was diluted 100-fold to obtain a concentration of 10^6^ CFU mL−1. Ciprofloxacin and meropenem were dissolved in caMHB, and 100 μL of each antibiotic solution were added and serially diluted into the 96-well plates (concentrations ranged from 32 ug/ml - 0.015625 ug/ml) followed by the addition of the same volume of bacterial suspension, resulting in a final bacterial density of 5 × 10^5^ CFU/mL. The microwell plates were incubated at 37 °C for 24 h, and growth was evaluated by eye. Triplicates were performed for each concentration. Bacteria alone or uninfected media were used as positive and negative controls, respectively. The experiment was repeated three times, and the highest MIC value obtained was reported.

**Supplementary figures**

***Fig.S1.* CONSORT Diagram.** *Of 34 assessed individuals, 6 were excluded, and 28 were randomized (14 active, 14 placebo). By Week 4, 2 placebo participants had discontinued (1 AE, 1 noncompliance), leaving 26 with available data (14 active, 12 placebo). By Week 12, a further 3 participants had dropped out (1 active due to noncompliance; 2 placebo due to AE and noncompliance), giving 23 completers (13 active, 10 placebo). Week 12 primary efficacy analyses were performed on the per‑protocol population (n = 23), with intention‑to‑treat sensitivity analyses for the primary outcomes.*

***Fig.S2.Serum Biomarkers****. Log2 Fold Change after 4 and 12 weeks in relation to baseline of Serum Biomarkers in Active and Placebo Groups.* ***a****, Systemic Inflammation.* ***b****, Gut Permeability.* ***c****, Intestine, Kidney and Endothelial function.* ***d****, Metabolism.* ***e****, Nutrition.* ***f****, Cell Death. Stars show p-value <0.5, Mann-Whithney U-test, alternative = less, comparing with the other arm at the same week.*

***Fig.S3.Fecal Biomarkers****. Log2 Fold Change after 4 and 12 weeks in relation to baseline of Fecal Biomarkers in Active and Placebo Groups. Stars show p-value <0.5, MannWhithney U-test, alternative = less, comparing with the other arm at the same week.*

(c)	(d)

***Fig.S4. Secondary Bile Acids****. Log2 Fold Change after 4 and 12 weeks in relation to baseline of Secondary Bile Acids, in Active and Placebo Groups.* ***a****, Secondary Bile acids measured in serum samples,* ***b****, Secondary Bile acids measured in fecal samples. Stars show p-value <0.5, Mann-Whithney U-test, alternative = less, comparing with the other arm at the same week.*

***Fig.S5.Targeted Metabolomics****. Log2 Fold Change after 4 and 12 weeks in relation to baseline of Targeted Metabolomic measured metabolites in Active and Placebo Groups. Stars show p-value <0.5, Mann-Whithney U-test, alternative = less, comparing with the other arm at the same week.*

***Fig.S6. Minimum inhibitory concentrations (MICs) of meropenem and ciprofloxacin against planktonic Klebsiella pneumoniae.***

***Fig.S7. Scatter plots showing abundance over time for species differentially abundant between Placebo and Treatment arms at week 12 using Mann-Whitney U-test and p<0.05.***

*Each panel reports the p-value and coefficient using a Mixed with Time and Treatment Arm as fixed effect, and Subject as Random effect. The coefficient represents the effect of Placebo on abundance. Species with estimated p-value < 0.05 are marked with an asterisk *, none remained significant after FDR correction.*

***Fig.S8. Scatter plots showing abundance over time for Virulence Factors differentially abundant between Placebo and Treatment arms at week 12 using Mann-Whitney U-test and p<0.05.***

*Each panel reports the p-value and coefficient using a Mixed with Time and Treatment Arm as fixed effect, and Subject as Random effect. The coefficient represents the effect of Placebo on abundance. Virulence Factors with estimated p-value < 0.05 are marked with an asterisk *, none remained significant after FDR correction.*

***Fig.S9. Scatter plots showing abundance over time for Antimicrobial Resistance Genes differentially abundant between Placebo and Treatment arms at week 12 using Mann-Whitney U-test and p<0.05.***

*Each panel reports the p-value and coefficient using a Mixed with Time and Treatment Arm as fixed effect, and Subject as Random effect. The coefficient represents the effect of Placebo on abundance. Antimicrobial Resistance Genes with estimated p-value < 0.05 are marked with an asterisk *, none remained significant after FDR correction.*

**Supplementary Table Titles**

*Supplementary Table S1. Patient Characteristics*

*Supplementary Table S2. Measured Biomarkers*

*Supplementary Table S3. Meteor-counting report*

*Supplementary Table S4. MSP Abundance table*

*Supplementary Table S5. Abundance table of genes grouped by KO annotation.*

*Supplementary Table S6. Gene abundance table of genes with homology to Virulence Factors.*

*Supplementary Table S7 Virulence Factors Differential Abundance*

*Supplementary Table S8. Correlations of Virulence Factors vs. Serum Biomarkers*

*Supplementary Table S9. Gene abundance table of Genes annotated as Antimicrobial Resistance Genes*

*Supplementary Table S10. Antimicrobial Resistance Genes Differential Abundance*

*Supplementary Table S11. Gene abundance table annotated Secondary Bile Acid Genes*

*Supplementary Table S12. Measured Faecal Secondary Bile Acids*

*Supplementary Table S13. Measured Serum Secondary Bile Acids*

*Supplementary Table S14. Metabolomics*

**Supplementary Table S1. Patient Characteristics***

|  | **Placebo (N=12)** | **Active (N=14)** |
| --- | --- | --- |
| Demographic Data |  |  |
| Age, median (range), years | 58.5 (35-75) | 58.5 (47-68) |
| Male sex, No. (%) | 9 (75.0) | 10 (71.4) |
| Body mass index, median (range), kg/m² | 27.8 (22.9-32.8) | 26.3 (20.4-32.8) |
| Liver Disease Severity |  |  |
| Child-Pugh score, median (IQR) | 7 (7-8) | 7 (7-8) |
| MELD score, median (IQR) | 13.2 (10.2-16.1) | 12.6 (9.7-13.5) |
| History of Decompensation, No. (%) |  |  |
| Ascites | 8 (66.7) | 11 (78.6) |
| Hepatic encephalopathy | 5 (41.6) | 2 (14.3) |
| Varices | 2 (16.7) | 6 (42.9) |
| Alcoholic hepatitis | 2 (16.7) | 0 (0.0) |
| Laboratory Parameters |  |  |
| Total bilirubin, median (range), μmol/L | 31 (19-40) | 41 (17-68) |
| Albumin, median (range), g/L | 34 (30-38) | 34 (30-39) |
| Creatinine, median (range), μmol/L | 65 (53-83) | 70 (60-81) |
| Sodium, median (range), mmol/L | 136.5 (134-137) | 137.2 (134-140) |

*Table adapted from: Liu J et al, Gut. 2024 Jun 6;73(7):1183-1198.

**Reference**

[1] Riva A, Gray EH, Azarian S, et al. Faecal cytokine profiling as a marker of intestinal inflammation in acutely decompensated cirrhosis. JHEP Rep 2020; 2:100151.

[2] Costea PI, Zeller G, Sunagawa S, et al. Towards standards for human fecal sample processing in metagenomic studies. Nat Biotechnol 2017; 35:1069-1076.

[3] Byrska-Bishop M, Evani US, Zhao X, et al. High-coverage whole-genome sequencing of the expanded 1000 Genomes Project cohort including 602 trios. Cell 2022; 185:3426-3440.e19.

[4] Ewels P, Magnusson M, Lundin S, et al. MultiQC: summarize analysis results for multiple tools and samples in a single report. Bioinformatics 2016; 32:3047-3048.

[5] Criscuolo A, Brisse S. AlienTrimmer: a tool to quickly and accurately trim off multiple short contaminant sequences from high-throughput sequencing reads. Genomics 2013; 102:500-506.

[6] Wen C, Zheng Z, Shao T, et al. Quantitative metagenomics reveals unique gut microbiome biomarkers in ankylosing spondylitis. Genome Biol 2017; 18:142.

[7] Li J, Jia H, Cai X, et al. An integrated catalog of reference genes in the human gut microbiome. Nat Biotechnol 2014;32(8):834-841.

[8] Le Chatelier E, Nielsen T, Qin J, et al. Richness of human gut microbiome correlates with metabolic markers. Nature 2013;500(7464):541-546.

[9] Plaza Oñate F, Le Chatelier E, Almeida M, et al. MSPminer: abundance-based reconstitution of microbial pan-genomes from shotgun metagenomic data. Bioinformatics 2019; 35:1544-1552.

[10] Fan Y, Støving RK, Berreira Ibraim S, et al. The gut microbiota contributes to the pathogenesis of anorexia nervosa in humans and mice. Nat Microbiol 2023; 8:787-802.

[11] Ruppé E, Ghozlane A, Tap J, et al. Prediction of the intestinal resistome by a three-dimensional structure-based method. Nat Microbiol 2019; 4:112-123.

[12] Bengtsson-Palme J, Hartmann M, Eriksson KM, et al. METAXA2: improved identification and taxonomic classification of small and large subunit rRNA in metagenomic data. Mol Ecol Resour 2015; 15:1403-1414.

[13] Chen L, Yang J, Yu J, et al. VFDB: a reference database for bacterial virulence factors. Nucleic Acids Res 2005;33: D325-328.

[14] Das P, Marcisauskas S, Ji B, et al. Metagenomic analysis of bile salt biotransformation in the human gut microbiome. BMC Genomics 2019; 20:517.
