# Supplementary material for: Impact of YAQ001 on the Gut Microbiome, Permeability and Markers of Systemic Inflammation in Patients With Cirrhosis
Source: Liver Int. 2026 Oct 21;46(11):e70933. doi: 10.1111/liv.70933 (PMC13686283; doi:10.1111/liv.70933)
Supplement: Supplementary file 1 [file LIV-46-0-s001.zip › liv70933-sup-0001-FigureS7@Revised figS7_msp_abundance_over_time.pdf]

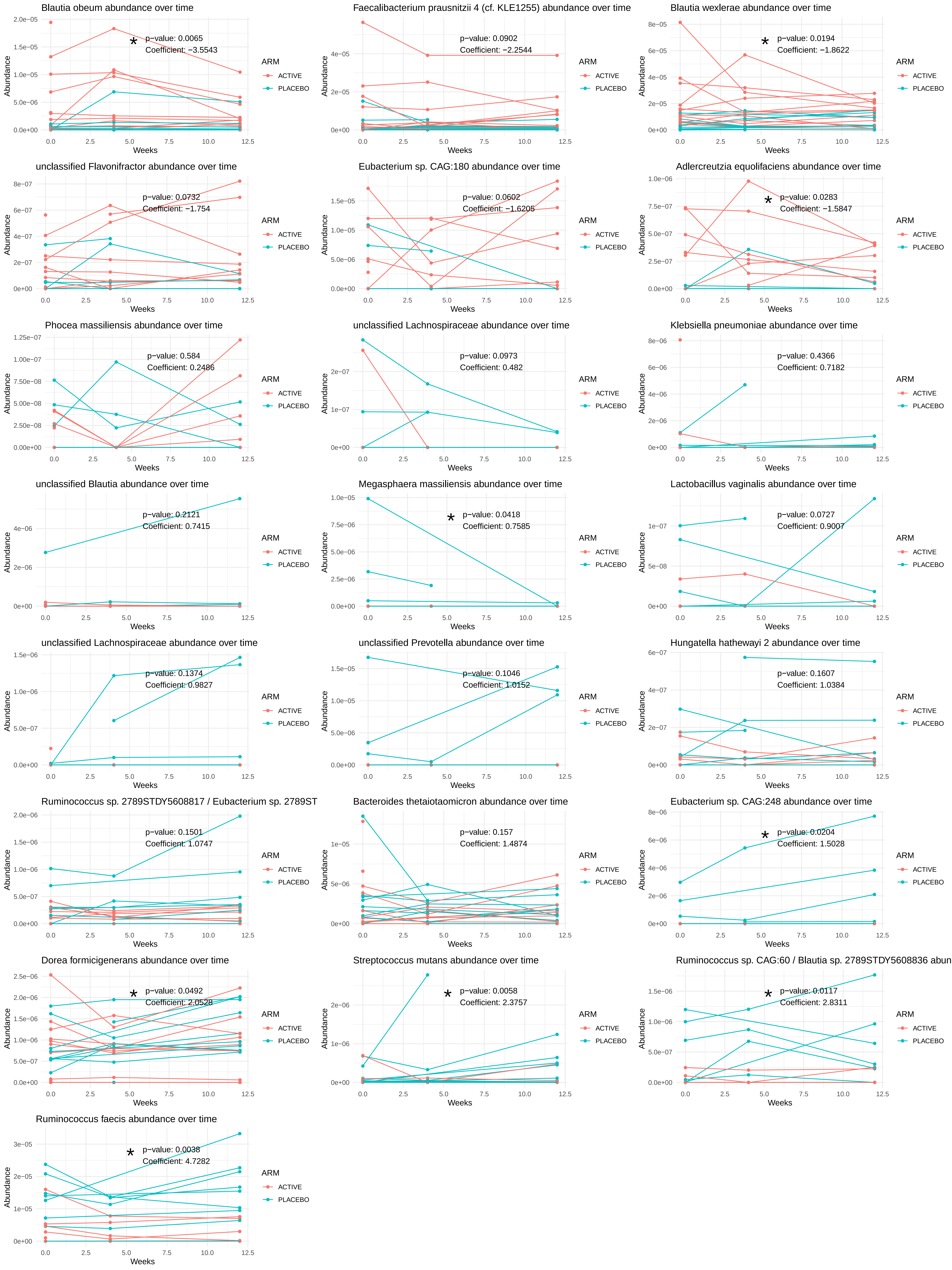
Blautia obeum abundance over time
p-value: 0.0065
Coefficient: −3.5543
Faecalibacterium prausnitzii 4 (cf. KLE1255) abundance over time
p-value: 0.0902
Coefficient: −2.2544
Blautia wexlerae abundance over time
p-value: 0.0194
Coefficient: −1.8622
unclassified Flavonifractor abundance over time
p-value: 0.0732
Coefficient: −1.754
Eubacterium sp. CAG:180 abundance over time
p-value: 0.0602
Coefficient: −1.6205
Adlercreutzia equolifaciens abundance over time
p-value: 0.0283
Coefficient: −1.5847
Phocea massiliensis abundance over time
p-value: 0.584
Coefficient: 0.2486
unclassified Lachnospiraceae abundance over time
p-value: 0.0973
Coefficient: 0.482
Klebsiella pneumoniae abundance over time
p-value: 0.4366
Coefficient: 0.7182
unclassified Blautia abundance over time
p-value: 0.2121
Coefficient: 0.7415
Megasphaera massiliensis abundance over time
p-value: 0.0418
Coefficient: 0.7585
Lactobacillus vaginalis abundance over time
p-value: 0.0727
Coefficient: 0.9007
unclassified Lachnospiraceae abundance over time
p-value: 0.1374
Coefficient: 0.9827
unclassified Prevotella abundance over time
p-value: 0.1046
Coefficient: 1.0152
Hungatella hathewayi 2 abundance over time
p-value: 0.1607
Coefficient: 1.0384
Ruminococcus sp. 2789STDY5608817 / Eubacterium sp. 2789ST
p-value: 0.1501
Coefficient: 1.0747
Bacteroides thetaiotaomicron abundance over time
p-value: 0.157
Coefficient: 1.4874
Eubacterium sp. CAG:248 abundance over time
p-value: 0.0204
Coefficient: 1.5028
Dorea formicigenerans abundance over time
p-value: 0.0492
Coefficient: 2.0528
Streptococcus mutans abundance over time
p-value: 0.0058
Coefficient: 2.3757
Ruminococcus sp. CAG:60 / Blautia sp. 2789STDY5608836 abun
p-value: 0.0117
Coefficient: 2.8311
Ruminococcus faecis abundance over time
p-value: 0.0038
Coefficient: 4.7282
Abundance
Weeks
ARM
ACTIVE
PLACEBO
