# Supplementary material for: Impact of YAQ001 on the Gut Microbiome, Permeability and Markers of Systemic Inflammation in Patients With Cirrhosis
Source: Liver Int. 2026 Oct 21;46(11):e70933. doi: 10.1111/liv.70933 (PMC13686283; doi:10.1111/liv.70933)
Supplement: Supplementary file 1 [file LIV-46-0-s001.zip › liv70933-sup-0002-FigureS8@Revised figS8_vf_abundance_over_time.pdf]

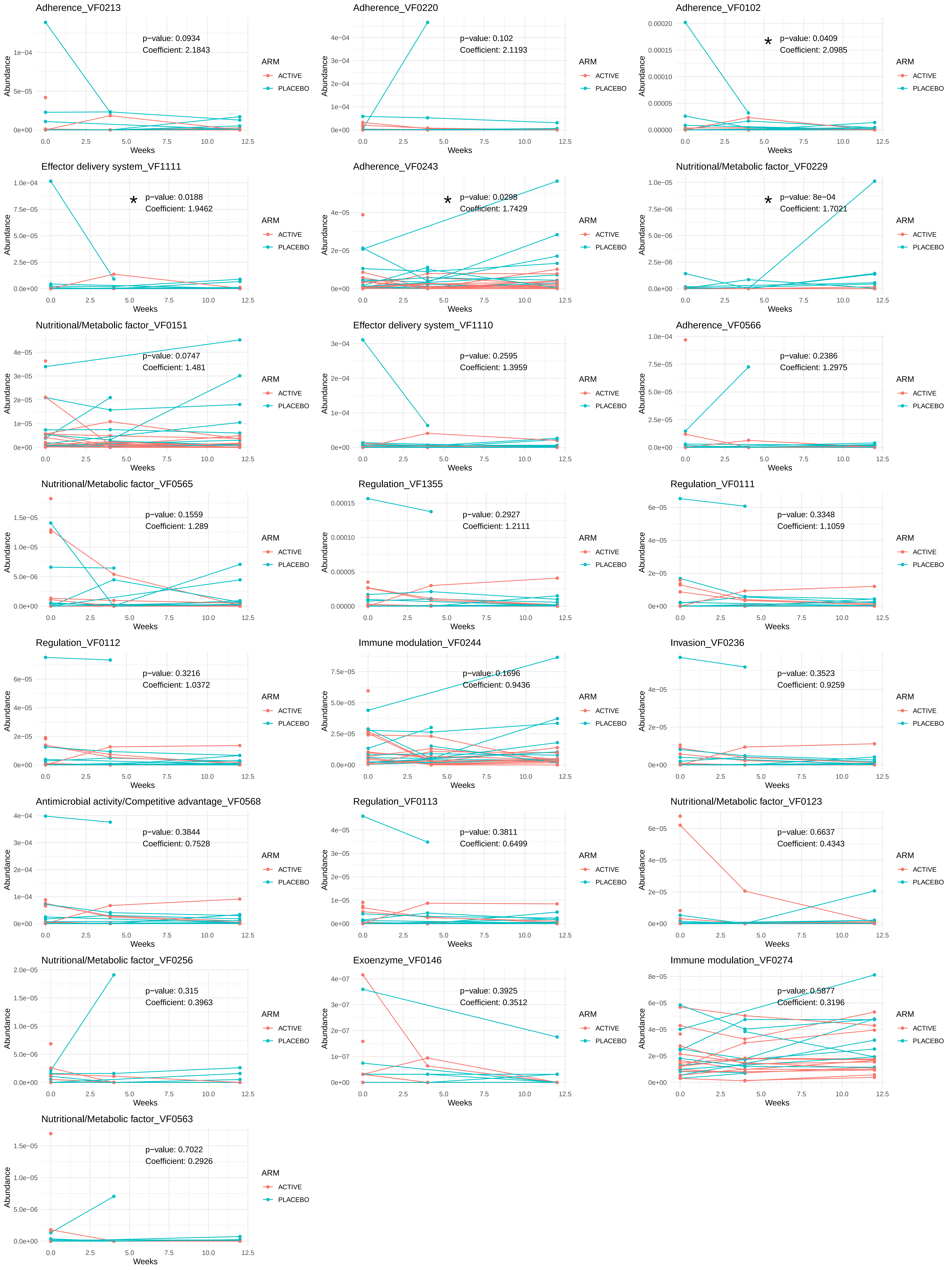

Adherence_VF0213
p−value: 0.0934
Coefficient: 2.1843
Adherence_VF0220
p−value: 0.102
Coefficient: 2.1193
Adherence_VF0102
*
p−value: 0.0409
Coefficient: 2.0985
Effector delivery system_VF1111
*
p−value: 0.0188
Coefficient: 1.9462
Adherence_VF0243
*
p−value: 0.0298
Coefficient: 1.7429
Nutritional/Metabolic factor_VF0229
*
p−value: 8e−04
Coefficient: 1.7021
Nutritional/Metabolic factor_VF0151
p−value: 0.0747
Coefficient: 1.481
Effector delivery system_VF1110
p−value: 0.2595
Coefficient: 1.3959
Adherence_VF0566
p−value: 0.2386
Coefficient: 1.2975
Nutritional/Metabolic factor_VF0565
p−value: 0.1559
Coefficient: 1.289
Regulation_VF1355
p−value: 0.2927
Coefficient: 1.2111
Regulation_VF0111
p−value: 0.3348
Coefficient: 1.1059
Regulation_VF0112
p−value: 0.3216
Coefficient: 1.0372
Immune modulation_VF0244
p−value: 0.1696
Coefficient: 0.9436
Invasion_VF0236
p−value: 0.3523
Coefficient: 0.9259
Antimicrobial activity/Competitive advantage_VF0568
p−value: 0.3844
Coefficient: 0.7528
Regulation_VF0113
p−value: 0.3811
Coefficient: 0.6499
Nutritional/Metabolic factor_VF0123
p−value: 0.6637
Coefficient: 0.4343
Nutritional/Metabolic factor_VF0256
p−value: 0.315
Coefficient: 0.3963
Exoenzyme_VF0146
p−value: 0.3925
Coefficient: 0.3512
Immune modulation_VF0274
p−value: 0.5877
Coefficient: 0.3196
Nutritional/Metabolic factor_VF0563
p−value: 0.7022
Coefficient: 0.2926
Abundance
Weeks
ARM
ACTIVE
PLACEBO
