# Supplementary material for: Impact of YAQ001 on the Gut Microbiome, Permeability and Markers of Systemic Inflammation in Patients With Cirrhosis
Source: Liver Int. 2026 Oct 21;46(11):e70933. doi: 10.1111/liv.70933 (PMC13686283; doi:10.1111/liv.70933)
Supplement: Supplementary file 1 [file LIV-46-0-s001.zip › liv70933-sup-0003-FigureS9@Revised figS9_arg_abundance_over_time.pdf]

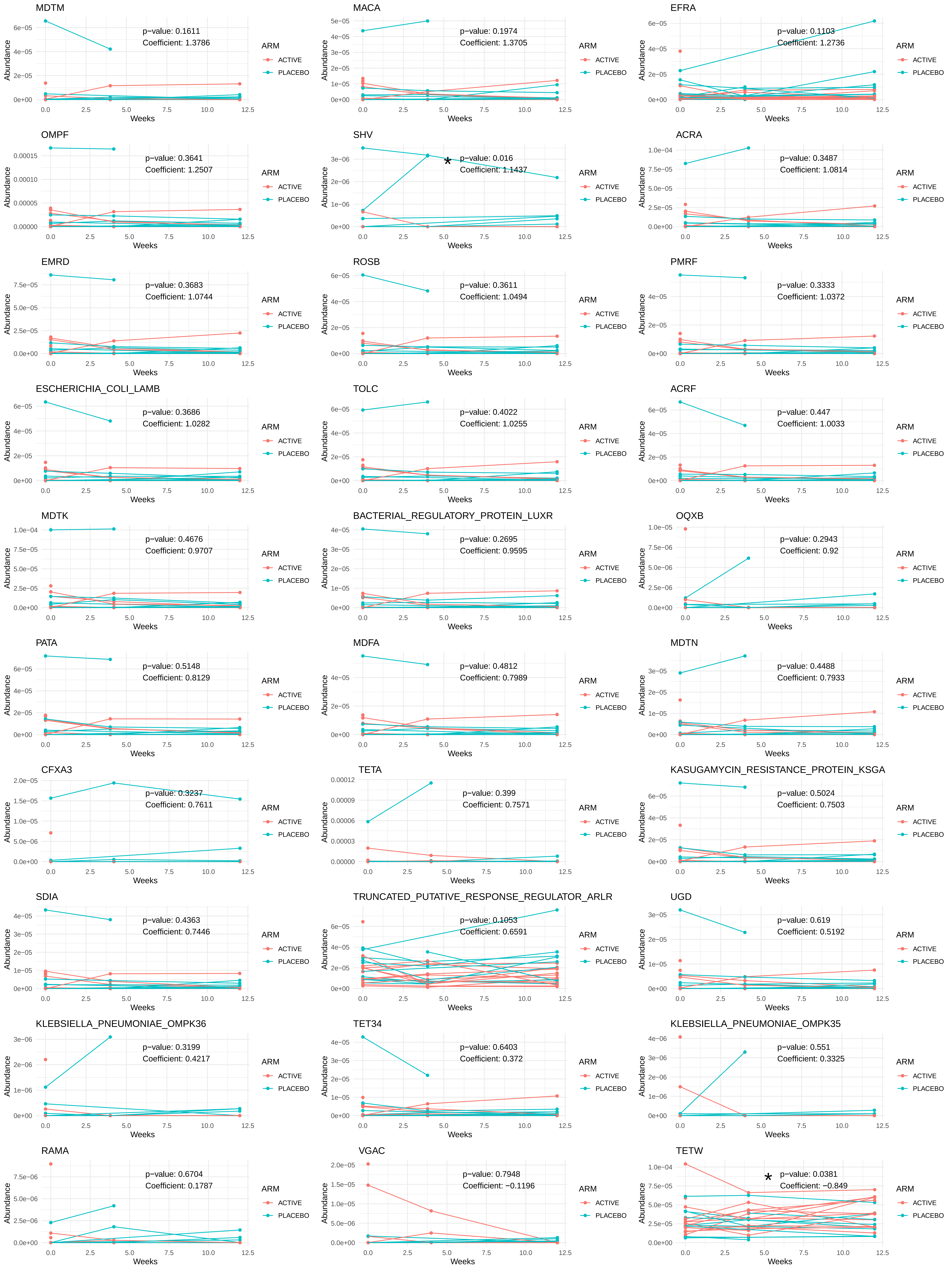
MDTM
p−value: 0.1611
Coefficient: 1.3786
MACA
p−value: 0.1974
Coefficient: 1.3705
EFRA
p−value: 0.1103
Coefficient: 1.2736
OMPF
p−value: 0.3641
Coefficient: 1.2507
SHV
*
p−value: 0.016
Coefficient: 1.1437
ACRA
p−value: 0.3487
Coefficient: 1.0814
EMRD
p−value: 0.3683
Coefficient: 1.0744
ROSB
p−value: 0.3611
Coefficient: 1.0494
PMRF
p−value: 0.3333
Coefficient: 1.0372
ESCHERICHIA_COLI_LAMB
p−value: 0.3686
Coefficient: 1.0282
TOLC
p−value: 0.4022
Coefficient: 1.0255
ACRF
p−value: 0.447
Coefficient: 1.0033
MDTK
p−value: 0.4676
Coefficient: 0.9707
BACTERIAL_REGULATORY_PROTEIN_LUXR
p−value: 0.2695
Coefficient: 0.9595
OQXB
p−value: 0.2943
Coefficient: 0.92
PATA
p−value: 0.5148
Coefficient: 0.8129
MDFA
p−value: 0.4812
Coefficient: 0.7989
MDTN
p−value: 0.4488
Coefficient: 0.7933
CFXA3
p−value: 0.3237
Coefficient: 0.7611
TETA
p−value: 0.399
Coefficient: 0.7571
KASUGAMYCIN_RESISTANCE_PROTEIN_KSGA
p−value: 0.5024
Coefficient: 0.7503
SDIA
p−value: 0.4363
Coefficient: 0.7446
TRUNCATED_PUTATIVE_RESPONSE_REGULATOR_ARLR
p−value: 0.1053
Coefficient: 0.6591
UGD
p−value: 0.619
Coefficient: 0.5192
KLEBSIELLA_PNEUMONIAE_OMPK36
p−value: 0.3199
Coefficient: 0.4217
TET34
p−value: 0.6403
Coefficient: 0.372
KLEBSIELLA_PNEUMONIAE_OMPK35
p−value: 0.551
Coefficient: 0.3325
RAMA
p−value: 0.6704
Coefficient: 0.1787
VGAC
p−value: 0.7948
Coefficient: −0.1196
TETW
*
p−value: 0.0381
Coefficient: −0.849
Abundance
Weeks
ARM
ACTIVE
PLACEBO
